# Supplementary material for: Identification of photoperiod-induced specific miRNAs in the adrenal glands of Sunite sheep (Ovis aries)
Source: Front Vet Sci. 2022 Jul 22;9:888207. doi: 10.3389/fvets.2022.888207 (PMC9354845; doi:10.3389/fvets.2022.888207)
Supplement: Supplementary file 1 [file Data_Sheet_1.ZIP › Supplementary file/miRDeep2 file.zip!/miRDeep2 file/Know_Structure/SPLP42A3/oar-mir-376d.pdf]

miRBase precursor : oar-mir-376d
Total read count : 60
oar-miR-376d read count : 58
remaining reads : 2

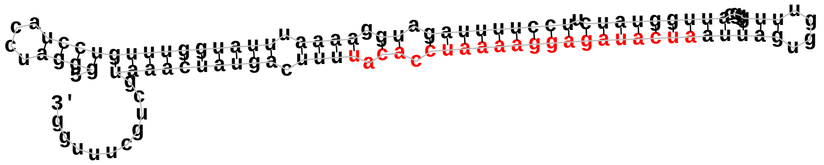

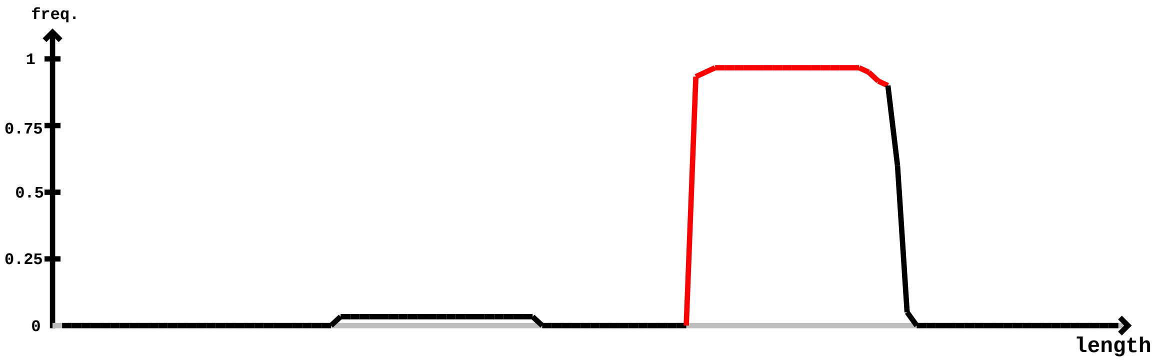

oar-miR-376d

```
5'- gggauccauccuguuugguauuuaaaagguagauuuuccuucuaugguuacggguuuggugauuaaucauagaggaaaauccacauuuucaguaucaaaugcugcuuugg   -3'    exp
    ((((....))))((((((((((.((((.((.(((((((((.(((((((((...(((....)))))))))))))))))))))).)).)))).)))))))))).........    reads      mm         sample
    ............................guagauuuuccuucuaugguu............................................................    2          0          seq
    .................................................................aucauagaggaaaaucca..........................    1          0          seq
    .................................................................aucauagaggaaaauccac.........................    2          0          seq
    .................................................................aucauagaggaaaauccaca........................    1          0          seq
    .................................................................aucauagaggaaaauccacau.......................    18         0          seq
    .................................................................aucauagaggaaaauccacauu......................    33         0          seq
    .................................................................aucauagaggaaaauccacauuu.....................    1          0          seq
    ..................................................................ucauagaggaaaauccacauuu.....................    1          0          seq
    ...................................................................cauagaggaaaauccacauuu.....................    1          0          seq
```
